# Lyme neuroborreliosis in Japan: *Borrelia burgdorferi* sensu lato as a cause of meningitis of previously undetermined etiology in hospitalized patients outside of the island of Hokkaido, 2010–2021

**DOI:** 10.1111/ene.70005

**Published:** 2025-01-14

**Authors:** Masayuki Ohira, Ai Takano, Kentaro Yoshi, Akira Arai, Yashuhiro Aso, Rikiya Furutani, Tadanori Hamano, Ikuko Takahashi‐Iwata, Chikako Kaneko, Tohru Matsuura, Norihisa Maeda, Hideto Nakajima, Katsuro Shindo, Toshihiko Suenaga, Kazuma Sugie, Yasuhiro Suzuki, Toru Yamashita, Frederick J. Angulo, Juanita Edwards, Cody Matthew Bender, Lisa R. Harper, Yoshikazu Nakayama, Shuhei Ito, Andreas Pilz, James H. Stark, Jennifer C. Moïsi, Hidehiro Mizusawa, Masaki Takao

**Affiliations:** ^1^ Department of Clinical Laboratory and Internal Medicine National Center of Neurology and Psychiatry Tokyo Japan; ^2^ Department of Veterinary Medicine, Joint Faculty of Veterinary Medicine Yamaguchi University Yamaguchi Japan; ^3^ National Research Center for the Control and Prevention of Infectious Diseases Nagasaki University Nagasaki Japan; ^4^ Department of Neurology Aomori Prefectural Central Hospital Aomori Japan; ^5^ Department of Neurology Oita Prefectural Hospital Oita Japan; ^6^ Department of Neurology National Hospital Organization, Shinshu Ueda General Hospital Nagano Japan; ^7^ Department of Neurology University of Fukui Hospital Fukui Japan; ^8^ Department of Neurology Hokkaido University Hospital Hokkaido Japan; ^9^ Department of Neurology Southern Tohoku General Hospital Fukushima Japan; ^10^ Division of Neurology Jichi Medical University Tochigi Japan; ^11^ Department of Neurology National Hospital Organization Beppu Medical Center Oita Japan; ^12^ Department of Neurology Nihon University Itabashi Hospital Tokyo Japan; ^13^ Department of Neurology Kurashiki Central Hospital Okayama Japan; ^14^ Department of Neurology Tenri Hospital Nara Japan; ^15^ Department of Neurology Nara Medical University Hospital Nara Japan; ^16^ Department of Neurology National Hospital Organization Asahikawa Medical Center Hokkaido Japan; ^17^ Department of Neurology Okayama University Hospital Okayama Japan; ^18^ Vaccines and Antivirals Medical Affairs, Pfizer Vaccines Collegeville Pennsylvania USA; ^19^ Vaccines Medical Affairs Pfizer Japan Inc Tokyo Japan; ^20^ Vaccines and Antivirals Medical Affairs, Pfizer Vaccines Vienna Austria; ^21^ Vaccines and Antivirals Medical Affairs, Pfizer Vaccines Cambridge Massachusetts USA; ^22^ Vaccines and Antivirals Medical Affairs, Pfizer Vaccines Paris France; ^23^ Department of Neurology National Center of Neurology and Psychiatry Tokyo Japan

**Keywords:** epidemiology, disease burden, Lyme neuroborreliosis, meningitis, tick‐borne disease

## Abstract

**Background and Purpose:**

Clinical manifestations of Lyme borreliosis (LB), caused by *Borrelia burgdorferi* sensu lato (Bbsl), include erythema migrans, Lyme neuroborreliosis (LNB), carditis, and arthritis. LB is a notifiable disease in Japan with <30 surveillance‐reported LB cases annually, predominately from Hokkaido Prefecture. However, LB, including LNB, may be under‐diagnosed in Japan since diagnostic tests are not readily available. We sought to determine if LNB could be a cause of previously undiagnosed encephalitis or meningitis in Japan.

**Methods:**

Investigators at 15 hospitals in 10 prefectures throughout Japan retrieved serum and/or cerebrospinal fluid (CSF) samples collected in 2010–2021 from 517 patients hospitalized with encephalitis or meningitis which had an etiology that had not been determined. Samples were tested for Bbsl‐specific antibodies using ELISA and Western blot tests. In alignment with the European Union LNB case definition, a confirmed LNB case had CSF pleocytosis and intrathecal production of Bbsl‐specific antibodies and a probable LNB case had a CSF sample with pleocytosis and Bbsl‐specific antibodies.

**Results:**

LNB was identified in three hospitalized patients with meningitis of previously undetermined etiology: a male resident of Aomori Prefecture was a confirmed LNB case, and two female residents of Oita Prefecture were probable LNB cases. None of the patients with confirmed or probable LNB had traveled in the month prior to symptom onset and none had samples previously tested for LB.

**Conclusion:**

The identification of previously undiagnosed LNB cases indicates a need for enhanced disease awareness in Japan, particularly beyond Hokkaido Island, and more readily available LB diagnostic testing.

## INTRODUCTION

Lyme borreliosis (LB) is an infection caused *Borrelia burgdorferi* sensu lato (Bbsl) which is transmitted to humans by *Ixodes* ticks [1]. The most common clinical manifestation of LB is erythema migrans, but Bbsl infection can disseminate and result in Lyme neuroborreliosis (LNB). Bbsl infections in Japan are caused by the genospecies *B. afzelii*, *B. bavariensis*, and *B*. *garinii*, which are transmitted by *I. persulcatus* [2, 3]. *I. persulcatus* is found on Hokkaido Island and, less commonly, elsewhere in Japan [2].

Laboratory‐confirmed LB cases are statutorily notifiable in Japan using a case definition of a symptomatic patient with Bbsl identified in skin or cerebrospinal fluid (CSF) by culture, Bbsl genes identified in CSF by polymerase chain reaction (PCR), and/or Bbsl‐specific antibodies identified in serum by Western blot. LB cases have been reported from several prefectures, particularly Hokkaido [3, 4]. LB is likely under‐diagnosed in Japan since diagnostic tests are not readily available [5]. From 2010 to 2021, an average of 17 laboratory‐confirmed LB cases a year were reported in Japan, 56% of cases were from Hokkaido Prefecture (https://www.niid.go.jp/niid/ja/ydata/11529‐report‐ja2021‐20.html). Japan also conducts surveillance for acute encephalitis with approximately 500 cases reported annually, half of which are of unknown etiology [6].

In contrast to LB, there is no specific case definition for LNB in Japan. The European Centre for Disease Prevention and Control conducts surveillance for LNB using the European Union (EU) case definition (https://www.ecdc.europa.eu/en/all‐topics/eu‐case‐definitions) which defines a confirmed LNB case as a patient with CSF pleocytosis and intrathecal production of Bbsl‐specific antibodies, and a probable LNB case as a patient with CSF pleocytosis and evidence of Bbsl‐specific antibodies in CSF.

Neither LNB cases nor LB cases with neurological symptoms are commonly identified in Japan; only 9 (8%) of 113 LB cases diagnosed at a Hokkaido hospital from 1989 to 2004 showed neurologic symptoms [7]. We sought to determine if LNB was a cause of encephalitis or meningitis of previously undetermined etiology throughout Japan.

## METHODS

Investigators at 15 hospitals in 10 prefectures throughout Japan (Figure S1) reviewed medical records to identify patients >1 year‐of‐age hospitalized in 2010–2021 with encephalitis or meningitis of unknown etiology and for whom a serum or CSF sample stored at ≤ −20°C was available. Patient's medical records were reviewed for information on travel in the month prior to symptom onset. Patient's diagnostic testing, which varied between participating hospitals, was not collected. The study was approved by the medical ethics committees at National Center of Neurology and Psychiatry, Nagasaki University, Yamaguchi University, and participating hospitals.

Retrieved samples received at Nagasaki University, after aliquoting, were sent to Yamaguchi University for Bbsl‐specific antibody testing (Figure S2). Samples with adequate sample quantity were diluted (serum: IgG 1:500, IgM 1:100; CSF IgG 1:10, IgM 1:5) and tested using an ELISA with established interpretative criteria (Figure S3). The ELISA test was based on antigens from *B. garinii* HP3, *B. afzelii* NT28, *B. bavariensis* NT29, *B. miyamotoi* MYK4, and *Borrelia* sp. tHM16w isolated from ticks in Japan [3, 8, 9, 10].

Samples that were positive with the first ELISA test or did not have adequate quantity for ELISA testing were diluted (serum: IgG 1:100, IgM 1:50; CSF: IgG 1:20, IgM 1:10) and tested by the recomLine Borrelia IgG, IgM Western blot following the manufacturer's instructions (Mikrogen GmbH, Germany). The Western blot‐identified IgG and IgM antibodies against p18, p39, p41, p58, p100, OspA, OspC, and VlsE. CSF samples with ≥1 band on Western blot were tested by real‐time polymerase chain reaction (PCR) for borrelial 16S rRNA and conventional PCR for borrelial *fla*B gene [10]. If sufficient sample volume, Western blot‐positive serum, and CSF samples were also tested for total IgG with Invitrogen IgG (total) Human ELISA kit (BMS2091).

Western blot results were used to identify intrathecal production of Bbsl‐specific antibodies; after excluding the Western blot results against p41 and OspC because of potential cross‐reaction with other bacteria, patients with Western blot Bbsl‐specific antibody bands that identified in CSF but not identified in serum were defined as having intrathecal antibody production. A confirmed LNB case was defined as a patient with a CSF sample with pleocytosis (nucleated cell count >5/μL), Bbsl‐specific IgM antibodies in CSF identified by Western blot, and Bbsl‐specific intrathecal antibody production. A probable LNB case was defined as a patient with a CSF sample with pleocytosis and Bbsl‐specific IgM antibodies in CSF identified by Western blot but no available evidence of Bbsl‐specific intrathecal antibody production.

## RESULTS

Adequate residual serum and/or CSF was available from 517 patients (Table 1). The median number (range) of patients per hospital with residual serum and/or CSF samples was 35 patients (3–61). Of the 517 patients, serum was available from 320 (61.7%) and CSF from 427 (82.6%), for a total of 747 samples. Of the serum samples, 4 (1.2%) were ELISA IgG‐positive and 7 (2.2%) IgM‐positive. Of the CSF samples, 36 (8.4%) were ELISA IgG‐positive and 8 (1.9%) were IgM‐positive. No samples were positive by PCR analysis.

**TABLE 1:** Residual serum and cerebrospinal fluid samples tested, and Lyme neuroborreliosis cases, among hospitalized patients with encephalitis or meningitis of previously undetermined etiology, by participating hospital, Japan, 2010–2021.

Hospital (Prefecture)	No. hospitalized patients with encephalitis or meningitis of previously undetermined etiology	No. (%) patients with residual sera available	No. (%) patients with residual CSF available	No. LNB cases
National Hospital Org. Asahikawa Medical Center (Hokkaido)	34	0 (0)	34 (100)	0
Hokkaido University Hospital (Hokkaido)	58	50 (86.2)	22 (37.9)	0
Aomori Prefectural Central Hospital (Aomori)	61	48 (78.7)	57 (93.4)	1^a^
Southern Tohoku General Hospital (Fukushima)	5	4 (80.0)	2 (40.0)	0
Jichi Medical University Hospital (Tochigi)	45	38 (84.4)	40 (88.9)	0
Nihon University Itabashi Hospital (Tokyo)	39	4 (10.3)	35 (89.7)	0
National Center of Neurology and Psychiatry (Tokyo)	3	3 (100)	3 (100)	0
National Hospital Org. Shinshu Ueda Medical Center (Nagano)	25	3 (12.0)	25 (100)	0
University of Fukui Hospital (Fukui)	13	13 (100)	0 (0)	0
Tenri Hospital (Nara)	10	7 (70.0)	6 (60.0)	0
Nara Medical University Hospital (Nara)	30	13 (43.3)	24 (80.0)	0
Okayama University Hospital (Okayama)	53	44 (83.0)	52 (98.1)	0
Kurashiki Central Hospital (Okayama)	46	33 (71.7)	39 (84.8)	0
National Hospital Organization Beppu Medical Center (Oita)	60	40 (66.7)	53 (88.3)	2^b^
Oita Prefectural Hospital (Oita)	35	20 (57.1)	35 (100)	0
Total	517	320 (61.9)	427 (82.6)	3

Abbreviations: CSF, cerebrospinal fluid; LNB, Lyme neuroborreliosis; Org, organization.

^a^ Confirmed.

^b^ Probable.

Three patients had Bbsl‐specitic antibodies in CSF on Western blot (Figure S4); one was confirmed and two were probable LNB cases (Table 2).

**TABLE 2:** Enzyme‐linked immunoassay and recomLine test results of Lyme neuroborreliosis cases retrospectively identified among hospitalized patients with encephalitis or meningitis of unknown etiology at 15 participating hospitals in Japan, 2010–2021.

Age years (sex)	CSF findings		ELISA (test result)^b^				Western blot antigens detected				Interpretation
	Nucleated cell count^a^	Total protein	Serum		CSF		Serum		CSF		
			IgG	IgM	IgG	IgM	IgG	IgM	IgG	IgM	
32 (M)	68/μL	100 mg/dL	0.0013 (Neg)	0.0003 (Neg)	0.2901 (Pos)	0.2744 (Pos)	Neg	p41	p41, p100, OspC	p39, p41, p100, OspC	Confirmed case
85 (F)	64/μL	893.1 mg/dL	0.0001 (Neg)	0.0007 (Neg)	QNS	QNS	p41	Neg	p41	p41, OspC	Probable case
35 (F)	567/μL	124.3 mg/dL	NA	NA	0.0513 (Pos)	0.1241 (Pos)	NA	NA	OspC	p41, OspC	Probable case

*Note*: ELISA serum IgG OD_(450–620)_ >0.2176 was IgG‐positive, and >0.1888 was IgM‐positive. ELISA CSF IgG OD_(450–620)_ >0.03864 was IgG‐positive, and >0.00927 was IgM‐positive.

Abbreviations: CSF, cerebrospinal fluid; dl, deciliter; ELISA, enzyme‐linked immunoassay; F, female; IgG, immunoglobulin G; IgM, immunoglobulin M; LNB, Lyme neuroborreliosis; M, male; mg, milligram; NA, sample not available; Neg, negative; Pos, positive; QNS, quantity not sufficient for testing; μL, microliter.

^a^ Pleocytosis defined as nucleated cell count >5/μL.

^b^ Invitrogen IgG (total) Human ELISA kit (BMS2091) results for the 32 year‐of‐age male were total IgG in serum 2550μg/mL and total IgG in CSF 18 μg/mL.

## DISCUSSION

Testing of the residual samples from >500 patients hospitalized with meningitis or encephalitis during an 11‐year period identified one confirmed and two probable LNB cases. Each of the LNB cases had CSF pleocytosis and Bbsl‐specific IgM antibodies identified in CSF by Western blot. Since there is not an LNB case definition in Japan, we used the EU LNB case definition and, to reduce the potential for a false positive detection of anti‐Bbsl antibodies [11], added the requirement that confirmed and probable LNB cases have Bbsl‐specific IgM antibodies in CSF detected by Western blot. Furthermore, we used Western blot test results to identify Bbsl‐specific intrathecal antibody production. In Europe, in contrast, most clinical laboratories use ELISA for LB diagnostic testing and the EU LNB case definition relies on ELISA test results to identify intrathecal antibody production.

Although CSF from three patients had pleocytosis and Western blot‐identified Bbsl‐specific IgM antibodies, intrathecal antibody production was confirmed in only one patient. Therefore, we identified one confirmed and two probable LNB cases. The first probable case had Western blot‐detected Bbsl‐specific p41 and OspC IgM antibodies in CSF but only p41 in serum. In our definition of intrathecal antibody production, we exclude Western blot results for p41 and OspC because of the potential for cross‐reaction [12]. The other probable LNB case had Western blot‐detected Bbsl‐specific p41 and OspC IgM antibodies in CSF, but a serum sample was not available.

The confirmed LNB case was a 32‐year‐old male Aomori City resident who was hospitalized with meningitis for 9 days in February 2021; Aomori City is in Aomori Prefecture, the northernmost prefecture of the Honshu Island, across the Tsugaru Strait from Hokkaido Island. The two probable LNB cases were Beppu City residents hospitalized in the same hospital; both began their hospitalization in May. One was an 85‐year‐old hospitalized for 35 days with encephalitis starting in May 2021, and the other was a 35‐year‐old hospitalized for 4 days with meningitis in May 2019. Beppu City is in Oita Prefecture, which is on Kyushu Island, >1000 kilometers south of Hokkaido. Each of the patients with LNB was fully recovered at the time of the discharge. None of the LNB patients received treatment for LB and none reported travel, tick bite, or exposure to tick habitat in the month prior to symptom onset.

A limitation of this retrospective study is that a serum and a CSF sample were available from three‐fifths and four‐fifths of patients, respectively. Furthermore, some of the available samples had an insufficient quantity for both ELISA and Western blot testing. If a serum and CSF sample of adequate quantity were available from all patients, more LNB cases would likely have been identified. There are limited data on circulating *Borrelia* strains in Japan. Samples in our study were screened with an ELISA test developed from *Borrelia* isolated from ticks in Japan; it is possible, therefore, that additional LNB cases may have been missed in this study if there are strains of *Borrelia* infecting humans in Japan that would not be detected by the in‐house ELISA and Western blot tests.

Despite not having an etiology determined for their meningitis or encephalitis, none of the patients in our study had been previously tested for LB. This may have been due to the low clinical suspicion for LB. [4] However, over 10% of patients, including the confirmed LNB case, resided in the northernmost part of Honshu Island, an area where LB cases had been reported previously (https://www.niid.go.jp/niid/images/epi/nesid/nesid_en.pdf). Another reason that LB was not tested for is likely that LB diagnostics are usually only available at reference laboratories [5].

LNB is an uncommon clinical manifestation of Bbsl infection. In Europe, it is estimated that approximately 5% of persons with untreated erythema migrans develop LNB [13]. Importantly, LNB can have serious disease with marked neurological deficits, and many patients have residual symptoms following hospital discharge [14]. Fortunately, LNB is treatable with appropriate antibiotics, emphasizing the importance of establishing an LNB diagnosis [15].

In conclusion, the identification of previously undiagnosed LNB cases in this study demonstrates that LB may be systematically under‐detected in Japan. These findings highlight the need for increased awareness, early and efficient clinical testing, and prompt treatment for Lyme disease in Japan as well as targeted prevention efforts to reduce LB risk.

## AUTHOR CONTRIBUTIONS

**Masayuki Ohira:** Conceptualization; investigation; methodology; project administration; software; supervision; writing – review and editing. **Ai Takano:** Conceptualization; data curation; formal analysis; investigation; methodology; project administration; software; supervision; validation; visualization; writing – original draft; writing – review and editing. **Kentaro Yoshi:** Conceptualization; data curation; investigation; methodology; project administration; software; supervision; validation; writing – review and editing. **Akira Arai:** Investigation. **Yashuhiro Aso:** Investigation. **Rikiya Furutani:** Investigation. **Tadanori Hamano:** Investigation. **Ikuko Takahashi‐Iwata:** Investigation. **Chikako Kaneko:** Investigation. **Tohru Matsuura:** Investigation. **Norihisa Maeda:** Investigation. **Hideto Nakajima:** Investigation. **Katsuro Shindo:** Investigation. **Toshihiko Suenaga:** Investigation. **Kazuma Sugie:** Investigation. **Yasuhiro Suzuki:** Investigation. **Toru Yamashita:** Investigation. **Frederick J. Angulo:** Conceptualization; data curation; formal analysis; methodology; project administration; resources; supervision; validation; visualization; writing – original draft; writing – review and editing. **Juanita Edwards:** Conceptualization; data curation; funding acquisition; methodology; project administration; resources; supervision; validation; writing – review and editing. **Cody Matthew Bender:** Data curation; methodology; writing – review and editing; formal analysis. **Lisa R. Harper:** Conceptualization; data curation; funding acquisition; methodology; project administration; resources; supervision; validation; writing – review and editing. **Yoshikazu Nakayama:** Conceptualization; methodology; validation; funding acquisition; writing – review and editing; project administration; data curation; supervision; resources. **Shuhei Ito:** Conceptualization; funding acquisition; methodology; project administration; resources; supervision; writing – review and editing. **Andreas Pilz:** Conceptualization; funding acquisition; methodology; writing – review and editing. **James H. Stark:** Conceptualization; funding acquisition; methodology; project administration; resources; supervision; writing – review and editing. **Jennifer C. Moïsi:** Conceptualization; methodology; funding acquisition; writing – review and editing; project administration; supervision; resources. **Hidehiro Mizusawa:** Conceptualization; investigation; methodology; project administration; supervision; writing – review and editing. **Masaki Takao:** Conceptualization; investigation; methodology; project administration; supervision; writing – review and editing.

## FUNDING INFORMATION

The study was conducted as a collaboration between National Center for Neurology and Psychiatry, Yamaguchi University and Pfizer. Pfizer funded this study and was involved in the study design, analysis, interpretation of data, writing of the report, and in the decision to submit the paper for publication.

## CONFLICT OF INTEREST STATEMENT

Lisa R Harper, Juanita Edwards, Cody Matthew Bender, Andreas Pilz, Shuhei Ito, Frederick J. Angulo, James H. Stark, Yoshikazu Nakayama, and Jennifer Moïsi are employees of Pfizer and may hold stock or stock options.

## Supporting information


Figure S1.

## Data Availability

Data available on request from authors.
