# Supplementary material for: Lyme neuroborreliosis in Japan: Borrelia burgdorferi sensu lato as a cause of meningitis of previously undetermined etiology in hospitalized patients outside of the island of Hokkaido, 2010–2021
Source: Eur J Neurol. 2025 Jan 14;32(1):e70005. doi: 10.1111/ene.70005 (PMC11729742; doi:10.1111/ene.70005)
Supplement: Supplementary file 1 — Figure S1. [file ENE-32-e70005-s001.pdf]

Supplementary figures

Lyme neuroborreliosis in Japan: *Borrelia burgdorferi* sensu lato as a cause of meningitis of previously undetermined etiology in hospitalized patients outside of the Island of Hokkaido, 2010−2021

Supplementary figure 1. Location of participating hospitals, including those with Lyme neuroborreliosis (LNB) cases, by prefecture in Japan. Participating hospitals with one or more LNB cases are shown with a red star.

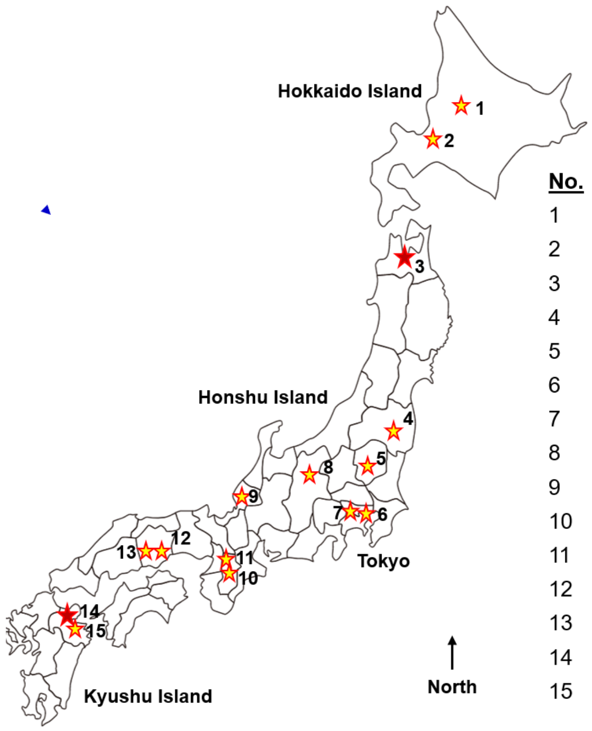

No.	Participating hospitals	Prefecture
1	National Hospital Organization Asahikawa Medical Center	Hokkaido
2	Hokkaido University Hospital	Hokkaido
3	Aomori Prefectural Central Hospital	Aomori
4	National Hospital Organization Southern Tohoku General Hospital	Fukushima
5	Jichi Medical University Hospital	Tochigi
6	Nihon University Itabashi Hospital	Tokyo
7	National Center of Neurology and Psychiatry	Tokyo
8	National Hospital Organization Shinshu Ueda Medical Center	Nagano
9	University of Fukui Hospital	Fukui
10	Nara Medical University Hospital	Nara
11	Tenri Hospital	Nara
12	Okayama University Hospital	Okayama
13	Kurashiki Central Hospital	Okayama
14	National Hospital Organization Beppu Medical Center	Oita
15	Oita Prefectural Hospital	Oita

Supplementary figure 2. Flow diagram of test procedures at Yamaguchi University laboratory

Samples received at Yamaguchi University with sufficient sample volume

↓

CSF samples examined for pleocytosis (nucleated cell count >5/µlx)

↓ Dilution

Screening test: ELISA

Serum: 1:500 (IgG) and 1:100 (IgM); CSF: 1:10 (IgG) and 1:5 (IgM)

↓

Specimens were tested with a Japanese ELISA test*, used by the Japan National Reference Laboratory, which used whole cell lysates of isolates from Japan (*B. garinii* HP3, *B. afzelii* NT28, *B. bavariensis* NT29, *B. miyamotoi* MYK4, and *Borrelia sp*. tHM16w)

↓

Using the cut-offs described in Supplementary Figure 3

Positive: serum with IgG OD$_{(450-620)}$ >0.2176 and/or IgM OD$_{(450-620)}$ >0.1888; CSF samples with IgG OD$_{(450-620)}$ >0.03864 and/or IgM OD$_{(450-620)}$ >0.00927

Using the cut-offs described in Supplementary Figure 3

Negative: serum with IgG OD $_{(450-620)}$ $\leq$0.2176 and IgM OD $_{(450-620)}$ $\leq$0.1888; CSF samples with IgG OD$_{(450-620)}$ $\leq$0.03864 and IgM OD$_{(450-620)}$ <0.00927

↓ Dilution (from Positive)

Confirmatory test: Western blot

Serum: 1:100 (IgG) and 1:50 (IgM); CSF 1:20 (IgG) and 1:10 (IgM)

↓

Western blot identified IgG and IgM antibodies against p18, p39, p41, p58, p100, OspA, OspC, and VlsE

↓

Western blot results used for:

1. Selection for polymerase chain reaction (PCR): CSF samples with >1 band on Western blot were tested by real-time PCR for borrelial 16S rRNA gene and conventional PCR for borrelial *flaB* gene.
2. Selection for testing for total IgG: Western blot positive samples tested with Invitrogen IgG (total) Human ELISA kit (BMS2091).
3. Identification of intrathecal production of Bbsl-specific antibodies (after excluding results against p41 and OspC): patients with Western blot Bbsl-specific antibody bands that were present in CSF but not present in serum were defined as having intrathecal antibody production.

↓

Confirmed LNB case: patient with a CSF sample with pleocytosis, Bbsl-specific IgM antibodies in CSF identified by Western blot, and Bbsl-specific intrathecal antibody production >0.00927.

Probable LNB case: a patient with a CSF sample with pleocytosis and Bbsl-specific IgM antibodies in CSF identified by Western blot but no available evidence of Bbsl-specific intrathecal antibody production.

*The Japanese ELISA test, which uses *Borrelia* strains isolated in Japan, is not available commercially. The Japanese ELISA test is more reliable than the commercial ELISA tests because commercial ELISA tests use *Borrelia* strains isolated in North America or Europe which may not be circulating in Japan. Bbsl, *Borrelia burgdorferi* sensu lato; CSF, cerebrospinal fluid; ELISA, enzyme-linked immunosorbent assay; IgG, immunoglobulin G; IgM, immunoglobulin M; OD, optical density; PCR, polymerase chain reaction; rRNA, ribosomal ribonuclear acid.

Supplementary figure 3. Procedure at Yamaguchi University laboratory for establishing thresholds for ELISA-positive samples (i.e., ELISA interpretative criteria) for ELISA (screening test)

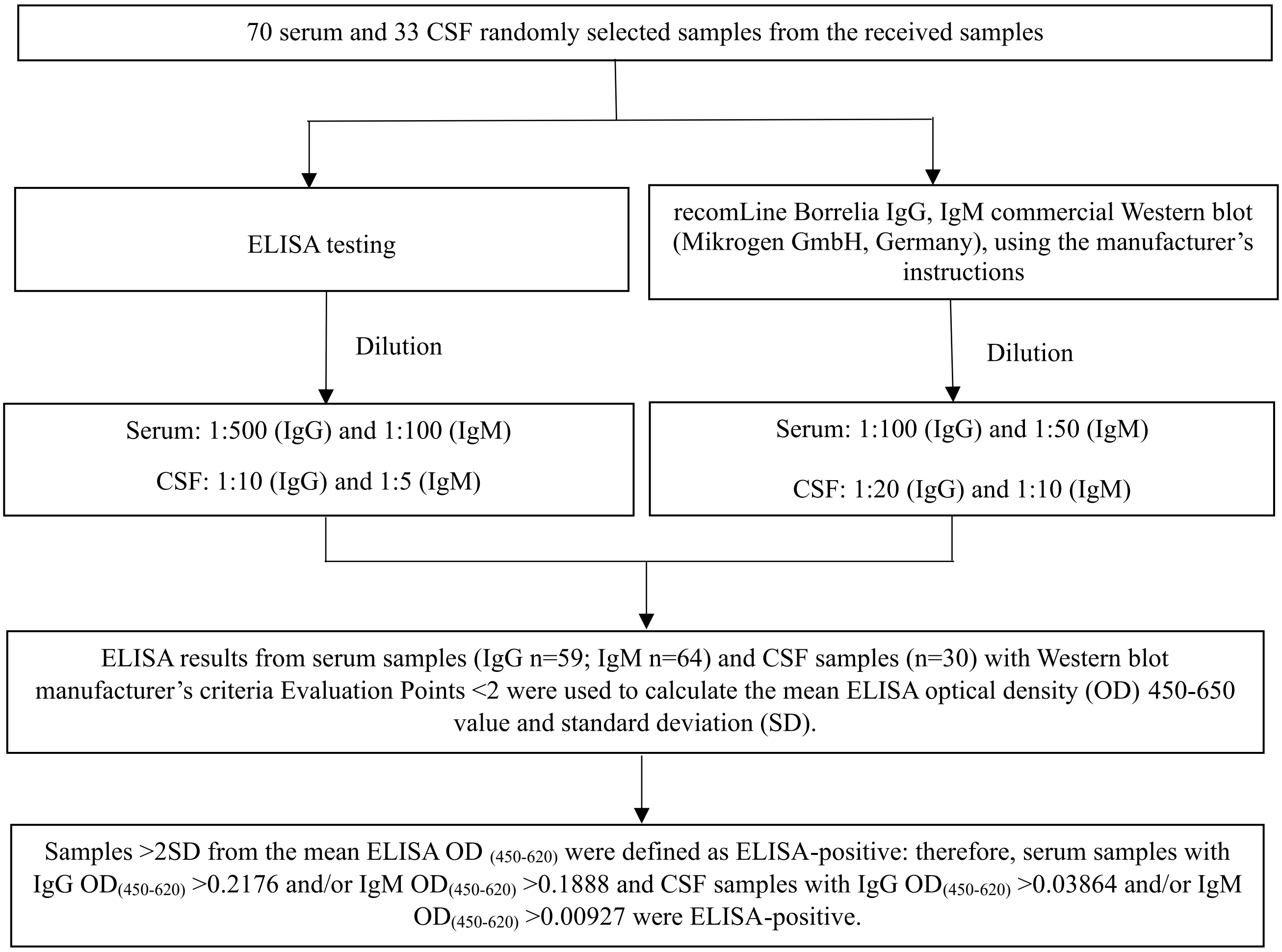

CSF, cerebrospinal fluid; ELISA, enzyme-linked immunosorbent assay; IgG, immunoglobulin G; IgM, immunoglobulin M; OD, optical density.

Supplementary figure 4. Results of Western blot testing to identify Bbsl- specific IgG and IgM antibodies in serum and CSF samples

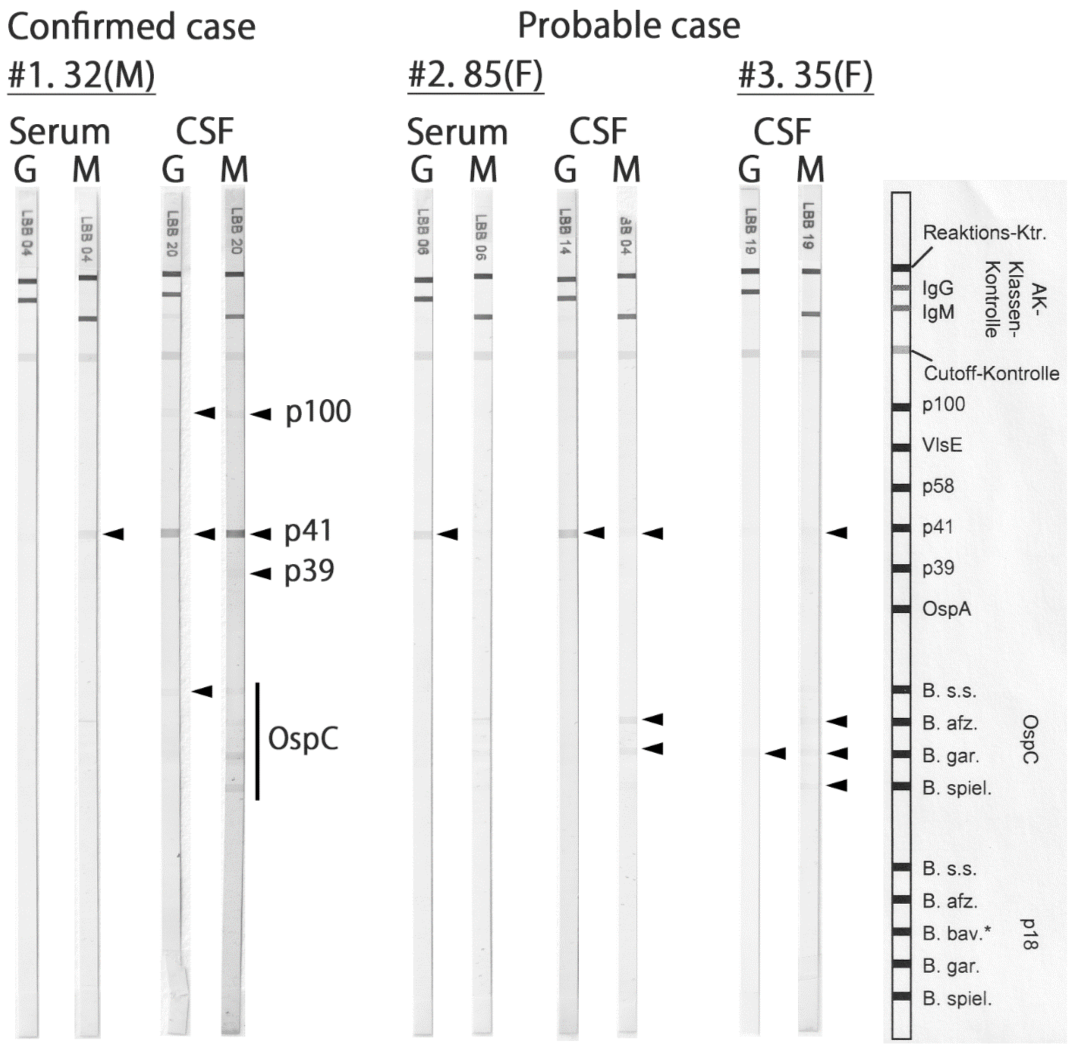

CSF, cerebrospinal fluid; G, Immunoglobulin G; M, Immunoglobulin; M, Male; F, Female
